# Barriers to Progress Feedback Adoption in Outpatient Geriatric Mental Healthcare: Exploring Age-Related Factors - A Qualitative Study

**DOI:** 10.1007/s10488-024-01402-1

**Published:** 2024-08-17

**Authors:** L. N. Frissen, P. D. Janse, R. V. Roskam, G. J. Hendriks

**Affiliations:** 1 Pro Persona Research, Gelderland, Netherlands; 2 Behavioural Science Institute, Radboud University, Nijmegen, Netherlands

**Keywords:** Geriatric mental healthcare, Progress feedback, Older adults, Implementation

## Abstract

Monitoring treatment progress through progress feedback is recognized for its efficacy and demonstrated value. However, its integration and utilization within treatments still need to be improved. Insufficient understanding exists regarding the factors within geriatric mental healthcare influencing the adoption of progress feedback. This study aimed to explore the determinants impacting the utilization of progress feedback within outpatient geriatric mental healthcare, specifically focusing on age-related perspectives and patient group characteristics. This qualitative investigation employed semi-structured interviews involving clinicians (*N* = 14) selected from four outpatient geriatric teams. The findings revealed both inhibiting and facilitating perspectives concerning progress feedback. Clinicians preferred user-friendly, specific, and tailored measures. Challenges included organizational support, integration in work processes, training, and the digital progress feedback system. Age-related perspectives such as older adults’ diverse issues, limited digital skills, and cognitive problems hindered implementation, particularly in the oldest generation of older patients. In outpatient geriatric mental healthcare, many factors and attitudes influencing progress feedback align with those observed in adult psychiatry literature. Moreover, this study highlights specific age-related factors that impede the adoption and implementation of progress feedback, shedding light on the specific barriers within this context.

## Introduction

Using progress feedback during psychological treatments can aid in tracking outcomes and evaluating the effectiveness of interventions (Barkham et al., 2023). Progress feedback, also referred to as Routine Outcome Monitoring (ROM) and Measurement-Based Care (MBC), involves measuring treatment progress systematically and discussing it using standardized questionnaires. This approach provides clinicians with insights into treatment stagnations that may go unnoticed by relying solely on intuition (Lambert, 2015). Various meta-analyses and reviews have shown that progress feedback has a small but positive impact on symptom reduction and reducing dropout rates (e.g., Lambert et al., 2018; De Jong et al., 2021; Rognstad et al., 2023). A recent review by Barkham et al. (2023) concludes that integrating progress feedback throughout different treatment phases is a cost-effective strategy to enhance outcomes and is particularly beneficial for patients not progressing as expected. Additionally, the use of progress feedback in treatment fosters improved communication between patient and practitioner (Carlier et al., 2012).

In recent years, substantial efforts have been directed towards implementing progress feedback, yet its practical application in treatment settings has been difficult. Low implementation rates of progress feedback have been observed across different age groups, including adults (e.g., Jensen-Doss et al., 2018) and youth (e.g., Van Sonsbeek et al., 2020), as well as in geriatric mental healthcare (Nuijen et al., 2015). In their review, Barkham and colleagues (2023) assert that successful implementation remains a significant challenge.

Qualitative studies can provide insight into reasons why therapists do or do not choose to use progress feedback, which in turn can help direct efforts to improve its implementation. Numerous qualitative studies concerning using progress feedback with adult clients have outlined factors influencing clinicians’ perspectives on progress feedback (Van Wert et al., 2021; Rye et al., 2019). Facilitating factors encompass easily accessible measurements, user-friendly interfaces, adequate training, organizational support, and the availability of relevant and valid instruments. Conversely, commonly cited barriers include time constraints, uncertainty about measurement selection, limited access to measurements, and inadequate training. Clinicians may exhibit negative attitudes towards progress feedback if they perceive it as not being suitable for patient groups with multiple and complex issues or if they believe that it may compromise the therapeutic relationship.

Despite the wealth of studies on progress feedback, research specifically exploring its use in geriatric mental healthcare remains sparse. Within geriatric health care, the Health of the Nation Outcome Scale For Elderly People (HoNOS65+) is an internationally used instrument to measure treatment outcomes. Its use is even mandatory in specialized mental healthcare in Australia and New Zealand. It can offer valuable insights into older patients’ psychological and social functioning (James et al., 2018), and scores on individual items help clinicians customize treatment to patients’ symptoms and assess treatment outcomes (Hermens et al., 2017; Veerbeek et al., 2013). However, it remains unclear whether the factors affecting the use of progress feedback in adult care also apply to geriatric mental healthcare or if there are additional unique aspects due to age-related and patient population characteristics.

Studies in geriatric mental healthcare underline that clinicians’ age-related perspectives shape treatment decisions. Research by Kessler and Schneider (2017) revealed that therapists-in-training working with older adults recommended shorter therapy durations, attributed less importance to motivational explanations, and deemed insightful techniques less suitable. Another study highlighted age-related prejudices impacting psychotherapy recommendations for older adults, resulting in less favorable attitudes, reduced willingness to provide treatment, and a more pessimistic prognosis (Kessler & Blanchetta, 2020). Moreover, amidst the COVID-19 pandemic, despite increased usage of digital health technology among older individuals, professionals often hold outdated perceptions regarding older people’s digital health skills (Mace et al., 2022).

These stereotypes, biases, and discriminatory attitudes based on age collectively constitute ageism, fostering erroneous assumptions and leading to prejudicial or discriminatory behaviors (World Health Organization, 2021). Recent systematic reviews indicate that ageism significantly contributes to poorer health outcomes worldwide. Alarmingly, healthcare professionals exhibit ageism tendencies, making decisions based on age stereotypes rather than the patient’s best interest (Chang et al., 2020). Given the widespread prevalence of ageism, this could also play a role in decisions regarding the use of progress feedback in older people.

By gaining a better understanding of the factors that impact progress feedback utilization within geriatric mental healthcare, particularly those related to age, we can improve implementation efforts. This knowledge can help us align progress feedback tools more effectively with clinical needs and support clinicians in their application. Ultimately, this could lead to improved treatment outcomes, reduced treatment duration, and stronger practitioner-patient collaboration.

In this qualitative study, we conducted semi-structured interviews with clinicians working in outpatient geriatric mental healthcare. Our aim was to unravel their experiences and viewpoints regarding progress feedback usage, specifically exploring the roles played by age-related factors and attitudes and how these factors influence progress feedback utilization.

## Method

### Research Design and Ethical Review

An exploratory qualitative study was conducted, consisting of semi-structured interviews with clinicians from four outpatient geriatric teams of a large mental health institution in the Netherlands. The research proposal was reviewed and approved by the Faculty of Social Sciences Ethics Committee of the Radboud University in Nijmegen (file code: ECSW-2021-100).

### Participants and Treatment Context

The participants in this study were 14 healthcare professionals working within one of the four specialized outpatient geriatric teams of a large Dutch mental health institution. Their disciplines included psychologists, psychiatrists, and nurses, representing the most important function groups within the teams. Participating clinicians also differed in age and the number of years of experience working within outpatient geriatric mental healthcare, having worked for at least one year in this field. See Table 1 for more information on the participants.

**Table 1:** Characteristics of participating clinicians

	*N*
*Team*	
A	3
B	5
C	3
D	3
*Discipline*	
Psychiatrist	3
Psychologist	6
Nurse	5
*Gender*	
Female	11
Male	3
*Age*	
21–30	3
31–40	4
51–60	5
61–70	2
*Experience in geriatric mental healthcare*	
0–5	7
6–10	4
11–15	1
16–20	1
41–45	1

Participants were recruited by sending the information letter and consent statement to all clinicians of the four geriatric teams by email and inviting them to respond. Because the response to the email was limited, potential participants were approached more specifically by location and job function.

During the recruitment and participant selection process, efforts were made to ensure a representative sample that mirrors the organization’s diverse job functions across the different teams. Attention was paid to variations in gender, age, discipline, and years of experience (Table 1).

Within the outpatient geriatric treatment teams, patients with diverse psychological problems (anxiety, depression, post-traumatic stress, psychosis, bipolar, and personality disorders) and who frequently also experienced somatic or mild cognitive problems were treated by a multidisciplinary team of clinicians. Evidence-based treatment methods were provided, such as Cognitive Behavioral Therapy, Acceptance and Commitment Therapy, EMDR, Schema Focused Therapy and pharmacotherapy.

In this healthcare institution, clinicians can select which questionnaires (commonly used self-report or clinician-administered questionnaires in mental health care) they want to utilize in a digital progress feedback system. For example, several teams in this mental health care institution used the Montgomery-Asberg Depression Scale to evaluate progress in the treatment of major depression. The questionnaire is sent to either the patient or the therapist, depending on the specific measure. For the geriatric teams, the digital feedback system automatically sends the HONOS65 + questionnaire to the healthcare professional at specific intervals. Clinicians are required to rate the functioning of each client using the 12 scales provided in this questionnaire. In addition to this standard feedback tool, clinicians have the freedom to choose and utilize their preferred questionnaires during treatment, allowing for variation in application and experiences. The scores from these questionnaires are displayed in the electronic patient record.

### Data Collection

All interviews took place online via video conferencing from October 2021 through February 2023. The duration of the interviews ranged from 30 to 60 min. The interview guide was based on knowledge derived from the literature review and the clinical experience of the first author in working in a geriatric team. Subsequently, the definitive interview guide was determined after the research team (consisting of this paper’s authors) reviewed it. It consisted of a set of key questions and a topic list to cover the same topics in all interviews. The key questions focused on the current use of progress feedback, experiences with this use, and their opinions about progress feedback. Specific questions were asked about age-related opinions and perceptions of the patient population characteristics and their role in using progress feedback. A pilot interview was conducted to test and adjust the interview. The interviews were recorded with an audio recorder and transcribed verbatim, omitting filler words and repetitions to promote readability. The audio files and anonymized transcripts were stored in a secure environment at the mental health institution following the General Data Protection Regulation (GPDR). Data collection occurred until no new information or themes emerged and content saturation was achieved. The coders evaluated saturation by analyzing data after each interview and continued this process until new interviews yielded redundant information. The results and saturation were then discussed with the entire research team. Achieving saturation indicated that further data collection was unlikely to provide additional insights, ensuring the study thoroughly explored the research question. Saturation was reached after 14 interviews.

### Data Analysis

Thematic analysis was used to analyze the collected data, applying the “Research Snake” model developed by Boeije and Bleijenbergh (2019). This cyclical method involves alternating between data collection and analysis. Three coding phases follow one another in this process: open coding (reading through data and developing a list of codes), axial coding (integrating codes around central categories), and selective coding (integrating findings by making connections between categories). To ensure the accuracy and consistency of the coding process, two coders independently coded the interviews, and their codes were subsequently compared. The program Atlas.ti version 23 was used for the data analysis.

## Results

During the qualitative analysis of the interviews, six themes emerged: (1) Age-related perceptions of older patients in general (2) Clinician’s attitudes towards progress feedback in general; (3) Feedback measures used in geriatric teams; (4) Age-related beliefs regarding the use of progress feedback; (5) Experiences using progress feedback within geriatric teams; (6) Clinician’s needs concerning progress feedback in general and specifically for older patients. The themes are illustrated with quotes from participants.

### Theme One: Age-related Perceptions of Older Patients in General

Cognitive problems, somatic frailty, and extensive comorbidity were common in the population they worked with, in the opinion of the clinicians. It was frequently mentioned that older adults have difficulty psychologizing (such as giving words to mental processes) and that giving a score to symptoms or states of mind is not natural for them. Clinicians found older adults more dependent on their clinicians, with less self-direction. They also mentioned greater vulnerability, reduced resilience workload, life-stage problems, difficult recovery, limited education, and socio-economic problems. Some clinicians felt older adults lacked digital proficiency in general, while others thought most were proficient.

A distinction was regularly made between the younger generation of older patients (aged 65 to 80) and the older generation of older adults (aged 80 and older), with the abovementioned age-related perceptions more frequently expressed in relation to the older generation.

Many older individuals with limited education have little experience with psychological language and, in general, with psychological processes and reflection. [resp. 1]

What I notice is that a small proportion [of older patients] is very digitally skilled, but the majority is not so digitally skilled. [resp. 10]

That people are more accommodating, to some extent. [They seem to have the attitude: ] You have to make me better. [resp. 11]

### Theme Two: Clinician’s Attitudes towards Progress Feedback in General

The theme includes both positive and negative attitudes toward progress feedback. Most respondents had a mixed view, seeing some aspects as helpful and others as obstructive.

#### Promoting Views

The most commonly shared promoting view was that progress feedback positively supports treatment. Respondents mentioned that it helps maintain focus, work methodically, provide direction, and end treatment. It adds value alongside clinical judgment, provides objective information, and structures the inquiry into symptoms. Clinicians were positive about using short, practical, and user-friendly questionnaires during treatment, which produced recognizable outcomes for patients. Some specifically mentioned the graph display of the digital progress feedback system as pleasant. Several clinicians regretted that feedback information was rarely used and felt opportunities still needed to be explored. There was interest in using feedback tools more frequently and extensively within treatment. Some clinicians were positive about using symptom-focused questionnaires, while others were positive about questionnaires that help monitor broad domains.

The use of Routine Outcome Monitoring helps keep a sharp focus on what you are treating. The measure also shows the difference. And then you can have a conversation about it. [resp. 7]

Because you can make certain things more transparent and not solely rely on your personal experience to say whether things are getting better or worse. [resp. 6]

#### Obstructive Views

Clinicians expressed concerns that the progress feedback did not always effectively support treatment or contribute value to clinical practice. This was attributed to the perception that some measures, such as the HoNOS65+, were too broad in scope. Many of them described progress feedback as an extra burden to an already heavy workload and felt it was cumbersome and time-consuming. Some experienced it as an obligation; they used it because the organization required it. Several clinicians felt that too much emphasis on measuring outcomes was not good, saw it as burdensome for the patient, and was impersonal. They experienced resistance to sending questionnaires digitally and strongly preferred conversing with the patient to evaluate treatment progress. A few clinicians mentioned uniformity and the association with benchmarking as hindering factors for using progress feedback.

I think you often assume, rightly or wrongly, that it won’t work. Or that it’s too much of a burden. And that, in any case, it will take more time and investment. [resp. 7]

Yes, HoNOS is too broad for me, it’s not helpful. It provides a general impression, but we already had that. I believe the health insurance company just uses that final measurement as a benchmark, and I’m absolutely against it. So, I don’t do that. [resp. 3]

### Theme Three: Feedback Measures used in Geriatric Teams

Clinicians described that in recent years, the HoNOS65 + was the main instrument used with all patients to measure the effect of treatment. At one site, the Montgomery Åsberg Depression Rating Scale (MADRS; Goekoop & Hartong, 2008) was also part of progress feedback in the treatment of mood disorders. The Montreal Cognitive Assessment (MoCA; Nasreddine et al., 2005; Dautzenberg & de Jonghe, 2004) or the Mini-Mental State Examination (MMSE; Folstein et al., 1975; Kok & Verhey, 2002) were also used when necessary to monitor cognitive functioning. Some clinicians also used symptom-focused questionnaires on a regular basis to monitor conditions such as depression or PTSD. Approximately half of the clinicians reported using the organization’s digital progress feedback system. The majority of clinicians indicated they did not actively use the HoNOS65 + in the sense that they did regularly fill in this questionnaire, but outcomes were rarely viewed and compared, discussed with patients and colleagues, and were rarely used to evaluate or adjust treatment. When symptom-focused questionnaires were used, outcomes were more frequently compared with previous results, discussed with patients and colleagues, and used to evaluate and adjust treatment. Some clinicians preferred to use paper questionnaires or had their own digital questionnaire files.

I don’t use the HoNOS; what I do instead is utilize questionnaires tailored for specific target groups. [resp. 3]

What I do use are questionnaires for specific target groups. So, for example, a depression questionnaire I use quite often. Just in order to monitor: how is it going with depression, and does my clinical impression match the rating on Hamilton, for example.” [resp. 3].

Except if you actively initiate it as a therapist, so I’ve initiated measurements for some clients, especially using the OQ-45 and occasionally the I.ROC. But you really need to take the initiative yourself to start that and ask the client to fill it out. [resp. 8]

### Theme Four: Age-related Beliefs Regarding the use of Progress Feedback

Specifically concerning progress feedback, some of the clinicians believed that it was neither possible nor useful with older adults, while others thought that the majority of older adults are perfectly capable of (digital) self-reporting, and this adds value to treatments.

The main obstacle to using progress feedback was the perception that older adults lacked sufficient digital proficiency. Many believed that older adults would struggle to fill in the measures, leading to underutilization of progress feedback. Difficulties in digital communication with older patients during clinical experiences also contributed to this perception. Some respondents particularly associated this barrier with the oldest group of older adult patients, while they believed that the younger generation of older people had sufficient digital skills to use progress feedback. Consequently, some clinicians actively initiated the use of progress feedback with the younger generation of older adults, while others did not change their approach. Clinicians also noted that the significant differences in digital skills among older adult patients complicated the implementation of progress feedback.

In addition to the perceived lack of (and wide disparity in) digital proficiency, other factors were identified as obstacles. Clinicians felt that the patient’s cognitive problems, unfamiliarity with measures, limited interest in progress feedback, and a preference for discussing progress instead of measuring it hindered the use of progress feedback and prevented them from using feedback themselves. Some clinicians indicated that most measures were not suitable for older adults in terms of content and hesitated to use progress feedback because they thought they were burdening patients. Some also said that their assessment of a patient’s overall vitality influenced their decision whether or not to use feedback.

The substantial variation in skills and problems in this patient group impeded the use of progress feedback because it was felt that every patient needed a different combination of questionnaires for them to be supportive of treatment. Another barrier was that adjustments were deemed necessary for a considerable part of the patient group to make progress feedback possible, such as support during completion, completing the questionnaire together during appointments, giving more time and explanation, using a paper version instead of a digital version, and involving the patient’s family.

Positive views on how clients perceive the use of progress feedback in their treatment facilitated the use of feedback.

I think I still see the difference between the new elderly and the old elderly who have little knowledge of computers. [resp. 9]

It’s often a complex picture, so I find it difficult to find a questionnaire that fits that. [resp. 4]

But no, I actually think that the idea that older adults are not yet digitized plays too big of a role in this. [resp. 2]

### Theme Five: Experiences Using Progress Feedback within Geriatric Teams

Positive experiences were related to its use on the client level. The experiences of discussing progress feedback with clients were positive, as was personalized progress feedback, in other words, using questionnaires tailored to the patient. A few clinicians also said they discussed the outcomes of measures during peer consultation or that it helped when colleagues reminded them to use a specific questionnaire. Some clinicians mentioned being accustomed to monitoring the course of the symptoms through other methods, such as the Life Chart to monitor mood, or frequently discussing a patient’s crisis management plan with them, reducing their need/tendency using questionnaires for feedback.

Negative experiences were mainly related to organizational factors. Clinicians felt that progress feedback was not integrated into the work processes. Also, although some clinicians said they had received instructions on how to use progress feedback, several said they had received little or no training in it and did not adequately know how to use the measures and digital progress feedback system. Psychologists indicated they the most knowledge on using progress feedback as monitoring symptoms and behaviors is often part of the treatment protocols they used.

Team-wide agreements on its use were not always clear. However, there were usually agreements about who was responsible for completing the questionnaires. There was no available overview of questionnaires suitable for older adults. Some clinicians indicated receiving the HoNOS65 + in large numbers at once, often not coinciding in time with actual patient appointments.

If I have measured outcomes and I have a peer consultation, I do discuss them then. [resp. 5]

I think that we actually do it too little, that it’s not sufficiently integrated into our workflow. [resp. 14]

It’s not facilitated that I know of. How do you fill it in, when, and which questionnaires for which disorder? Are there specific questionnaires for older adults? [resp. 6]

### Theme Six: Clinician’s Needs Concerning Progress Feedback in General and Specifically for Older Patients

First, needs were expressed that concerned using progress feedback in general. The most frequently expressed needs had to do with improving factors on the organizational level: its integration into working processes, better facilitation of its use, clear departmental policies, and team agreements. Further training and more mutual exchange with colleagues on this subject were desired, as was a flexible progress feedback system. There was a preference for short and quick questionnaires to administer or complete; practical usability was crucial. A reminder for administering and linking it to an appointment would be supportive.

Second, therapists expressed needs that were specifically concerned with using progress feedback with older adults. Regarding content, the needs diverged; some wanted more use of symptom-focused questionnaires, while others wanted questionnaires focused on multiple areas of life and problems common in older adults. There was a need for an overview of measures suitable for older adults, both within and outside the digital progress feedback system. Furthermore, therapists wanted a shift from using clinician-rated outcome measures (as is the case with the HoNOS65+) to using self-report instruments.

When it comes to those questionnaires, I think it should be something simple that takes into account a digital gap while still being serious. [resp. 10]

I think you can make it easier for a lot of colleagues. That you need to create much more policy around it. [resp. 13]

Due to the vast diversity among older adults, it’s no longer a ‘one size fits all’ approach. You really have to use elements and ingredients from the Routine Outcome Monitoring (ROM) in a very tailored way for each patient. [resp. 12]

## Discussion

This qualitative study explored the attitudes and experiences of clinicians regarding progress feedback within geriatric outpatient mental healthcare and investigated the role of age-related factors in the use of progress feedback.

The results showed that while most clinicians saw the added value of progress feedback in theory, they experienced barriers in using it in clinical practice, some of which were related to general views and experiences with progress feedback, while others related to specific views on using it with older adults. Clinicians were positive about progress feedback measures when they are user-friendly, measure specific symptoms, and can use questionnaires tailored to the patient instead of general questionnaires. However, many barriers were experienced as well. In line with previous studies on adults, clinicians point to the importance of organizational factors, such as integration in work processes, raising awareness about using progress feedback and promoting a feedback culture, training in using feedback, access to appropriate questionnaires, an adjustable digital progress feedback system, and their own opinions about progress feedback (Barkham et al., 2023; Van Wert et al., 2021; Rye et al., 2019, Trimbos Institute, 2015). Specific barriers concerning using progress feedback with older adults were related to age-related perceptions, such as the opinion that it is unsuitable for older adults due to the considerable variety of problems they experienced, which hindered its use and implementation. Views on, among other things, limited digital proficiency and cognitive problems in older adults deterred clinicians from using progress feedback. This was reinforced by experiences where these perceived characteristics of older adults gave problems in progress feedback use. It is noticeable that the majority of the clinicians, in their views about older adults, consistently introduced the nuance that it mainly concerned the oldest generation of older adults. The question is whether these beliefs about digital proficiency are justified or point to a possible bias. Recent survey studies show that in the US, UK, and Netherlands, more than 80% of 65-75-year-olds use the internet for various reasons. For those aged 75 years and older, this figure is approximately 50–60% (Pew Research Centre, 2024; Office of National Statistics, 2021; Statline, Centraal Bureau voor de Statistiek, 2023). Although this percentage is significantly lower than that of their younger counterparts, the number has been steadily increasing in recent years. This indicates that while older adults may require more assistance in learning how to use digital systems, they are willing and, to some extent, capable of using them.

Clinicians in the present study held familiar stereotypical views about older adults, as extensively described in the literature (Kessler & Schneider, 2017; Mace et al., 2022; World Health Organization, 2021; Chang et al., 2020). However the clinician’s perspectives were also influenced by their practical experience. Thus, the stereotypical views of clinicians seem to be both nuanced by the perceived diversity within the patient group, and at times, confirmed and possibly reinforced by the significant additional problems within that same patient group.

Even though no previous research has examined the role of these age-specific factors on the use of progress feedback, these findings are in line with research in which age-related factors have been found to influence treatment decisions (Kessler & Schneider, 2017; Kessler & Blanchetta, 2020). It is also in line with the conclusions drawn in the literature that clinician decisions are often made from (unconscious) ageism rather than what is best for the specific patient (Bodner et al., 2018, pp. 241–262; Chang et al., 2020). The time pressures often experienced within healthcare may further enhance the role of biases in medical decisions (Stepanikova, 2012).

In addition to age-related factors playing an impeding role in the use of progress feedback, many clinicians also had positive views about using progress feedback use with older adults. Nonetheless, as indicated by previous research, positive views toward progress feedback do not necessarily translate to increased utilization in adult mental health care settings (Rye et al., 2019). In the present study, specific organizational choices may have played a role in its utilization. The organization had included the HoNOS65 + in the standardized feedback system for several years, but the questionnaire was not considered practical or useful in clinical practice. Consequently, it was underutilized by clinicians. This, in turn, did not apply to symptom-oriented questionnaires, which people used on their own initiative because they experienced their added value. Staff members with negative experiences and opinions regarding the HoNOS65 + nonetheless see the added value of progress feedback within treatment if instruments better suit their needs.

## Strengths and Limitations

As far as we know, this is the first qualitative study to examine the role of age-related factors and patient characteristics in using progress feedback within geriatric mental healthcare. Furthermore, to our knowledge, no previous study has examined which factors and opinions specifically play a role within geriatric mental healthcare concerning progress feedback. This study’s strength lies in its inclusion of psychologists, psychiatrists, and nurses from four different locations, providing a reasonably representative reflection of the outpatient geriatric teams within a large mental health institution. In addition, the study has several limitations. First, this study focused solely on clinicians’ experiences, while patients’ experiences and views on using progress feedback are equally important. As there has not been any previous research on how older adults experience progress feedback, this would be important to study in the future. Secondly, the generalizability of the findings to geriatric mental healthcare in general may be limited as the participants were solely from one organization (although from four different treatment sites). The attitudes towards progress feedback may differ among clinicians in other organizations.

## Recommendations

Future research should further explore the impact of age-related and patient-specific factors on the utilization of progress feedback in geriatric mental healthcare. This understanding can assist organizations and teams in adjusting their policies and practices. Comparing the views of users and non-users, examining the experiences of older adults themselves, and contrasting them with the perspectives of clinicians can provide valuable insights.

Although progress feedback has proven to be effective (e.g., De Jong et al., 2021), and most clinicians in this study indicated they see the possible added value of using it, some barriers to using it with older patients need to be addressed. At the organizational level, there’s a need to highlight the importance of progress feedback in treatment and foster a culture that encourages feedback. Clinicians should be supported in integrating feedback into their daily work processes, given time to become more familiar with its use, and provided with ongoing training on questionnaire utilization. Crucially, management should seek and utilize feedback from the workplace consistently to improve the facilitation of progress feedback.

Concerning the feedback system, it’s advisable to provide clinicians with an overview and information about appropriate questionnaires for older adults and specific patient groups, diagnoses, and treatment objectives. Additionally, the system should offer a flexible approach for tailored monitoring, logically link measurement moments to appointments and evaluations, and issue reminders for completion and discussion to both clinicians and patients.

At the clinician level, efforts should be made to enhance their knowledge of feedback, including its importance and usefulness, and raise awareness about ageism and prejudices related to older individuals. For example, clinicians should inquire about a patient’s digital skills instead of assuming whether they can or cannot complete digital assessments. If necessary, extra support should be provided. Lastly, at the client level, there’s a need to provide explanations about progress feedback and its role in shared decision-making during treatment. Moreover, addressing potential difficulties clients might encounter when completing questionnaires and collaboratively seeking solutions with clinicians is essential.
